# An Eye Tracking Investigation of Young People's Gaze Behaviour to Gambling and Non-Gambling Moving Adverts

**DOI:** 10.1159/000529114

**Published:** 2023-02-07

**Authors:** Tochukwu Onwuegbusi, Amanda Roberts, Stephen Sharman, Todd Hogue

**Affiliations:** ^a^School of Psychology, University of Lincoln, Lincoln, UK; ^b^National Addiction Centre, Kings College, London, UK

**Keywords:** Advertising, Gambling, Sports betting, Eye tracking, Group comparisons, Gambling craving

## Abstract

**Introduction:**

Data from several areas of public health (e.g., harmful alcohol and tobacco) are consistent with the assertion that children's exposure to advertising strategies increases intention to consume such products. Most studies have measured self-rated impact of gambling advertising using questionnaires. Given that gambling advertisements come in different forms such as print media/television advertising and contain variable content, it is difficult to understand using subjective measures which aspects of the gambling advertisements increase craving and desire to trigger a gambling session. In the present study, we applied a novel data-driven methodology that directly tracks eye movements to reveal attentional biases towards gambling adverts and promotions by examining differences in young people's eye gaze behaviour when watching gambling and non-gambling (control) moving adverts.

**Method:**

A total of 98 (16–18 years old) children who self-identify as having a low or high craving to gambling watched gambling and non-gambling (control) television adverts, while their eye movements were recorded.

**Results:**

The results show that the data-driven method can isolate video clips that best distinguish people on the low-high craving spectrum, reveal the type of each video clip with the largest group differences, and accurately predict young people's gambling craving on the basis of eye movement patterns.

**Conclusion:**

Our findings demonstrate that young people's craving for gambling can be predicted based on their eye movements to video clips of gambling advertisements and that certain features of gambling advertisements may be more appealing to some group of viewers, particularly those with high craving for gambling.

## Introduction

The UK is one of the few jurisdictions where there are gambling forms that are legal and actively promoted for children. For instance, children of any age can gamble legally on category D machines, which are low-stake fruit-style machines, coin pushers (sometimes called penny falls), or crane grabs and can be located in the following places (e.g., casinos, betting shops, tracks with pool betting, bingo premises, adult gaming centres) [1]. The annual Young People and Gambling Survey conducted in 2020 by Ipsos MORI on behalf of the Gambling Commission showed that 7% of 11–16 years old in England and Scotland have gambled in the last 12 months while 1.9% of the same age cohort were classified as “problem gamblers” based on the DSM-IV-MR-J screen [2]. Increased media exposure to gambling advertisements and promotions has a direct link to gambling attitudes and intentions [3, 4], particularly among young people and children [5]. In 2013, the UK Office of Communication [6] published their research showing that on British television (between 2006 and 2012), children under the age of 16 were exposed to an annual average of 211 gambling adverts [6] while young people are likely to experience gambling-related harms [7] due to strong exposure to gambling adverts [8, 9]. Children in the UK are exposed to gambling in myriad ways; in a recent UK study [10], children aged 11–24 reported television, high street shops, and social media as the most ubiquitous routes to exposure to gambling advertising (85%, 70%, and 66%, respectively). Exposure on social media was most likely to be in the form of video adverts while watching clips on YouTube or ads appearing while scrolling through Facebook feeds.

### Technology and Gambling

Rapid advancements in technology have created a more accessible gambling industry, and the rise in gambling participation is now becoming particularly prominent [11], among children and young people. The last decade has seen a dramatic change in children's media use due to a surge in the use of interactive/online computer games, mobile phones, and especially the internet [12]. Promoting gambling through media adverts may help normalise gambling behaviour among young viewers [13, 14]. This assertion was affirmed in a recent research, which showed that 41,000 UK followers of gambling-related accounts are likely to be under the age of 16 [10]. Advertising portrays gambling as a fun and thrilling with some promise of social and financial success, and attempts to engage youth via exciting colours, upbeat music, and classy graphics [15, 16]. However, the absence of counter or balanced messages means that young people may not be fully aware of the potential risks and negative consequences resulting from gambling or able to differentiate the persuasive intent of embedded gambling promotions [5].

### Gambling Motives

There is a growing body of evidence that suggests gambling adverts are a driving motive for increased gambling participation [17], which could be exacerbated as the adverts become more prevalent [18, 19]. For example, the 2018 FIFA World Cup was a watershed moment for the proliferation and visibility of gambling advertising in the UK's public awareness [20] with a total of 69 live-odds adverts displayed within advertisement breaks in 32 games of the FIFA World Cup [21]. Some scholars theorize that gambling adverts are effective because they exploit gamblers' vulnerable cognitive functions: cognitive appraisal (e.g., reward seeking and decision-making) and cognitive differences (e.g., self-perceived skill) [20, 22]. This is because gambling adverts are displayed with the purpose of increasing gambling craving, while portraying gambling as a socially acceptable behaviour [23, 24].

### Gambling Craving

The study of gambling cravings has received a growing interest over the past years and has enduring impact [25]. Evidence from meta-analytic [26] and literature [27] reviews has shown that gambling advertising induces cravings to gamble and reinforces gambling behaviour over time through the process of classical conditioning [28]. Studies have also shown that pathological gamblers often experience overwhelming cravings as a response to advertisement cues [29]. This is consistent with research on the role of marketing cues in maintaining addictive behaviours, which suggests that advertising cues may induce cravings, thereby facilitating higher purchase [30] and highly harmful consumption [31].

### Features of Gambling Advertising

Some studies have shown that individuals participate in gambling because of the high associated rewards [32]. However, recent qualitative research suggests that there are different themes and features of gambling advertising that attract the attention of children, young people, and vulnerable adults [10]. This study revealed aspects of adverts that participants particularly found appealing included the use of music, colours, characters, and celebrities, features that would have an obvious appeal to children and young people. However, it also demonstrated that children and young people are attracted to a wide range of other features that are not unique to their cohort. For instance, participants were attracted to advertising which reinforced the “fun” element of participating in gambling with low risks while gambling advertisements that used humour were perceived to have universal appeal to all children and adults [8].

### Surveying Advertising Impact on Gambling

Data from several areas of public health (e.g., harmful alcohol and tobacco) are consistent with the assertion that children's exposure to advertising increases intention to consume such products [33]. However, obtaining a reliable measure of exposure to gambling advertising has been extremely difficult for past researchers [34]. Most studies have measured self-rated impact of gambling advertising by means of questionnaires, individual interviews, and focus groups for adults [14, 35] and young people [16, 36, 37, 38]. Conclusions drawn from these studies suggest that both adults and young people are accustomed to gambling advertising; however, they hold a negative attitude towards advertising in general, which is seen as exaggerated, biased, and employing different psychological tricks to push people to gamble more [25]. These subjective measures are useful as they can provide a rich amount of information. The premise here is that individuals have some insights of their own behaviour. However, this is an issue because gambling advertising is a controversial topic and people may be disinclined to speak freely about their attitudes and perceptions [34]. Another issue with subjective measures in general is they are susceptible to nonresponse bias [39] and socially desirable responses [40]. Given that gambling advertisements come in different forms (i.e., print media/television advertising) and content, it is difficult to understand which aspects of the gambling advertisements increase craving and desire to trigger a gambling session using subjective measures. Besides, previous research has shown that peoples' self-assessment as to what extent their consumer behaviour is influenced by advertising is unreliable [34].

### Eye Tracking

Tracking eye movements, by contrast, in addition to being ecologically valid, importantly enables the investigation of attentional biases not only at stimulus onset but also during the entire duration of the stimulus presentation [41]. Tracking eye movements is an effective means of studying information intake [42] as well as in measuring people's overt attentional focus when performing social activities or visual task [43, 44, 45]. Traditionally, eye movement studies generate a rich amount of data, which normally are based on two measures, fixations and saccades, signifying where, when, and how the eyes gather information from the visual world. Importantly, analysis of saccades and the number and duration of fixations provide an index of the underlying cognitive mechanisms that guide eye gaze [46].

### Eye Tracking in Gambling and Advertising Studies

Few studies have applied eye tracking to the study of gambling advertising; for reviews, see [47]. Sandberg and colleagues [48] measured eye movements of 39 Swedish teenagers aged 15 years while browsing the internet for approximately 15 min. Of the 5,000 advertisements identified, 576 had gambling content. The teenage participants attended to ten percent of all potential advertisements and were largely unaware of this exposure. Overall, boys attended to gambling advertising three times more than the girls, suggesting gender differences in exposure. However, the use of still images curbs generalisability to other forms of dynamic gambling advertising such as those aired on different television channels, during or around the broadcast of sporting events, lasting between 30 s and 1 or 2 min. Also, dynamic adverts (e.g., TV adverts) can show things moving whereas still adverts cannot. From an eye tracking perspective, people may be more able to control their eye movements more closely for still than dynamic (moving) adverts. Thus, interesting group differences that occur for other parts of the stimuli may be missed using still images, assuming the researcher uses still adverts with defined regions of interest.

As another example, Lole and colleagues [49, 50] applied eye tracking to examine responsible gambling (RG) messages [49] and inducement offers [50], in the wider context of gambling advertisements. In both studies, regular sports bettors and non-gamblers were presented with series of televised sports betting advertisements while their eye movements were tracked. In the first study [49], there were differences in the number of fixations placed on different types of RG messages based on gambling risk and the physical features of the messages. Regular gamblers viewed RG messages less than the control. In the second study [50], more fixations were placed on reduced risk and cash back inducements compared to better odds and bonus bet independent of level of gambling risk. Although Lole et al. used different sports betting advertisements, which were recorded from actual television broadcasts, they only focused on inducement information; thus, interesting group differences that occur for other parts of the stimuli may be missed.

### Present Study Aims

In the present study, we undertake one of the first applications of a newly developed data-driven method for analysing eye tracking data for dynamic stimuli [51]. We use this in a sample of young people to examine whether it can reveal group differences in video clips depicting gambling advertisements. The aims are (1) to test whether we can identify differences in the eye movement patterns of 16–18 years old who self-identify as having a low or high craving to gambling and (2) to uncover the type of adverts that reveal such differences. As stimuli, we used a set of short video extracts showing gambling as a FUN, gambling as a SPORT, gambling product and non-gambling adverts (four adverts per category). Furthermore, (3) we applied the machine learning techniques to examine if we can predict young people's craving for gambling based on their eye movements to moving gambling adverts. Although we did not have strong a priori expectations regarding the types of advert clips that would yield the strongest group differences, we did expect that adverts depict gambling as FUN to reveal larger group differences in eye movement patterns between the high and low craving group. This is because when the fun element is in view, there may be little else for observers to avert their gaze and look at, resulting in large group differences. Our study will utilize a new eye tracking methodology to reveal whether this is indeed the case.

## Methods

### Participants

Using an opportunistic sampling method, a total of 98 students were recruited from two secondary schools in England (74 females, 24 males). Participants had an age range of 16–18 (*M* = 17.01, *SD* = 0.68) and most (92%) identified as White British. Of the 98 participants, 28 had never gambled before and 70 had gambled before. A total of 23 participants were of legal gambling age in the UK (18 years) compared to 75 who were not. Each participant received £5 shopping voucher as a recompense for participation. All participants had normal or corrected-to-normal vision.

### Design

A within-subject design was used. Each participant was shown a random sequence of 16 video (advert) clips (with a mixture of 4 video clips showing gambling as a FUN, gambling as a SPORT, gambling product and non-gambling adverts).

### Stimuli/Materials

#### Gambling Craving Scale

The Gambling Craving Scale (GACS) [52] is a 9-item multidimensional measure of gambling-related cravings. The GACS assesses three dimensions of gambling cravings: desire (e.g., “I need to gamble now”), anticipation (e.g., “If I had an opportunity to gamble right now, I would probably take it”), and relief (e.g., “Gambling would make me less depressed”). Responses are anchored on a 7-point Likert scale with options ranging from 1 “Strongly Disagree” to 7 “Strongly Agree.” Internal consistency has been reported in previous studies for the total scale, α = 0.89 and subscales: anticipation, α = 0.79; desire, α = 0.94; and relief, α = 0.77 [53]. In addition, Young and Wohl [52] reported good reliability for the three subscales: anticipation, α = 0.84; desire, α = 0.81; and relief, α = 0.85. Cronbach's alpha for the 9 items in the current sample was good (α = 0.83) (the mean and standard deviation by participants' gender in high and low craving group for total GACS and each of the subscales can be found in the online suppl. material; for all online suppl. material, see www.karger.com/doi/10.1159/000529114).

#### Advertisement

**Fig. 1:**
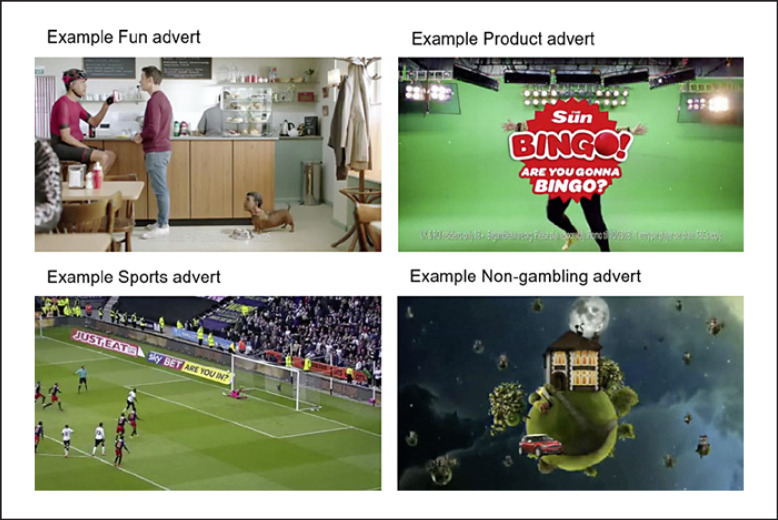
Example advert clips used in the study. Fun advert is an advert that reinforces the fun element of gambling. Sports type advertisement is an advert that depicts gambling as a sport. Product type advertisement is an advert that advertises a specific gambling product, such as a particular slot machine or table game. Non-gambling advert is an advert showing TV commercials (e.g., energy supplier and department store).

Sixteen different advertisements were used, sourced from YouTube and reduced in length using OpenShot (to comply with the fair use copyright policy for academic research). Each montage of adverts lasted around 30 s and showed past or current television gambling adverts and commercials (non-gambling content). There were 4 adverts (see Fig. 1) depicting gambling as fun (FUN element), 4 adverts depicting gambling as a form of sports (SPORTS element), 4 adverts showing gambling products (PRODUCTS element), and 4 randomly selected adverts showing TV commercials (e.g., energy supplier and department store).

### Apparatus

All the 16 video clips were presented on the 24-inch screen of a Tobii T60 XL eye tracker at a 1,280 × 900 video resolution and from a distance of around 65 cm. Eye movements in both eyes were tracked at a sampling rate of 60Hz. The Tobii T60 XL has a reported resolution of 0.5° and accuracy of 0.35° and applies both bright and dark pupil tracking. While the eye tracker automatically parses the recorded eye movements into fixations, saccades, and blinks, we used the eye movement recordings sampled at 60Hz, coding blinks as missing values.

### Procedure

All the participants were tested individually in a quiet, darkened room. They were asked to sit at a desk looking directly at the screen of the Tobii eye tracker with their chin in a chin rest positioned at 65 cm from the display. The Tobii's default 9-point calibration sequence was performed, involving participants fixating a series of nine red circles distributed across the screen. Next, participant's gaze point was presented as a circle in real time on the screen simultaneously with an image containing nine dots. Calibration was considered successful when the gaze point overlaps the dots. Viewing and recording were binocular. Following successful calibration, participants were provided with written instructions on the screen and were afterwards prompted to press a key to begin the experiment. Participants were shown the 16 video clips in succession, while their eye movements were recorded. After watching all the 16 video clips, they filled out the pen-and-paper questionnaires (GACS) and self-reported their age, gender, ethnicity, and how many times they have gambled in the past 1 week (a description of the participants who gamble vs. not in the past week, according to gender and ratings of craving, is shown in the online suppl. material). Following this, they were debriefed and thanked for their participation.

### Data Analysis

**Fig. 2:**
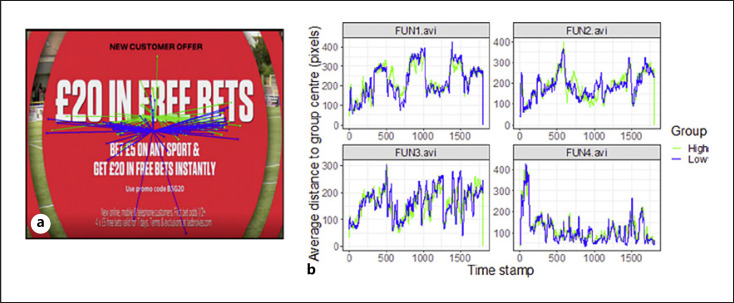
Illustration of the method. **a** Example of a video frame with superimposed gaze positions of the high craving participants (green dots) and low craving participants (blue dots). Lines connect the gaze position of each participant with their group centre. The method compares the average or summed length of the lines connecting the gaze positions to the group centre. **b** Average distance to group centres over time for low and high craving participants. Higher values indicate more variance in the gaze position within the group.

Figure 2a, b illustrates the method employed for data analysis. We applied a recently developed data-driven method for analysing eye tracking data for dynamic stimuli [51]. The authors identified two methods to quantify group differences in eye movement patterns: (1) separate comparisons of horizontal and vertical gaze position (comparing central tendency differences between groups − either horizontally or vertically, or both) and (2) comparisons of the distance to the group centres (comparing divergence difference between groups). The latter method, using the distance to group centre, was used to analyse the data in the current study. This method uses Student's *t* test to “test” for group differences. Whereas normally, such tests are used to uncover significant group differences, this was not our aim. Instead, we used the test as a means to uncover the type of video clips (adverts) that may be relevant for uncovering group differences (without drawing any conclusions about statistical significance). For the chosen method, the following processing steps were involved: (1) the horizontal and vertical gaze position was identified for a particular video frame, (2) gaze positions for each frame were compared between groups with Student's *t* tests by comparing the distance to the group centre, and (3) videos and types (sections) of videos were identified with large group differences. To isolate video clips with large differences in gaze behaviour between the two groups, we thus computed for each frame, participant, and video combination the average Euclidean distance (in pixels) to each of the group centres, thereby focusing on the dispersion in eye movements inside each group. We then computed the percentage of frames that showed a “significant” difference in these distances to the group centres using a Student's *t* test (uncorrected critical *p* value of 0.05). For scores on GACS questionnaire, a median split (median = 16) was used to split the group into high craving (*n* = 45) and low craving (*n* = 53) group.

### Machine Learning

**Fig. 3:**
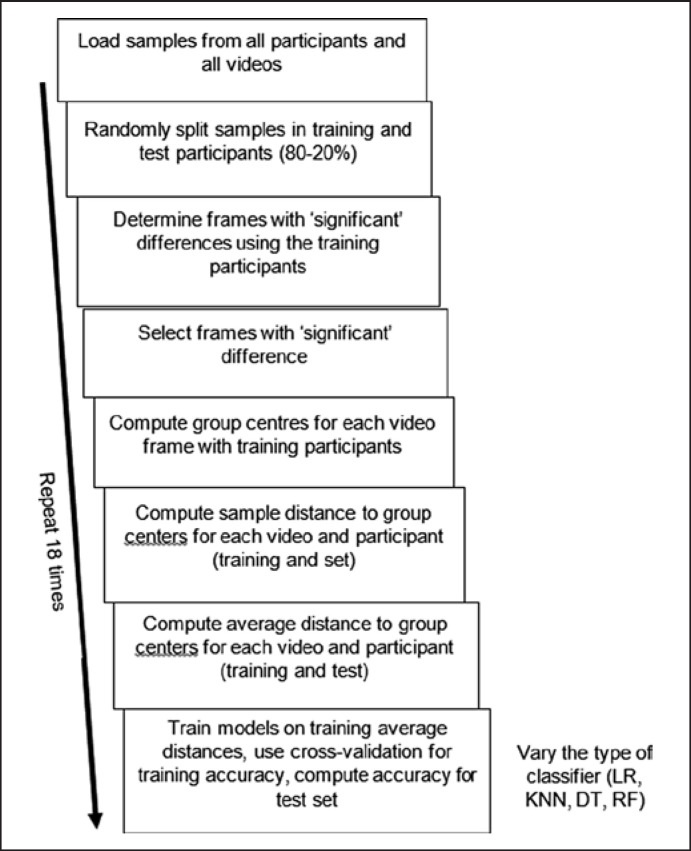
Steps for evaluating how well the proposed method predicts group membership. Throughout the process, training (random selection of 80% of participants) and test (remaining 20% of participants) are kept separately, so that the accuracy on the test participants would reflect performance if a new batch of participants would be classified with the method. The 18 repetitions of the process were used to determine how strongly the end results depend on the random split between training and test participants. The number of repetitions was a balance between computing time and sufficient information about the average performance and variability.

To examine whether we could distinguish between groups on the basis of the raw eye movement data, we used machine learning algorithms. Because we were unsure which algorithm would work best, we tested a variety of methods: logistic regression, k-nearest neighbour (KNN), decision tree, and random forest (RF) (an ensemble method that is less sensitive to correlated predictors) [54, 55]. We employed R's caret package [54] using the default parameters of the various models. In this paper, we present the results based on the distance towards the group centres. To examine how well new participants (unseen data) would be classified, we split data into a training set (80% of participants) and test set (20% of participants). The test set was set aside, videos and frames of videos were selected, and machine learning models were trained with the training set. The participants in the test set (yet unseen by the model) were then classified with the trained model to determine how well new participants can be classified on the basis of their eye movements (similar to when the test would be used for diagnostics). The processing steps involved in evaluating the model are shown in Figure 3. Because of the number of participants in the current study and a single split of the data in a training and test set could reflect the random split of the data to some extent, we relied on multiple random splits of the original dataset into training and test sets and computed the average performance across these multiple random splits. Performance was evaluated for the test set (we used accuracy) and the training set (where we used a 5-fold cross-validation) [56].

## Results

### Identification of Frames with a Difference

**Fig. 4:**
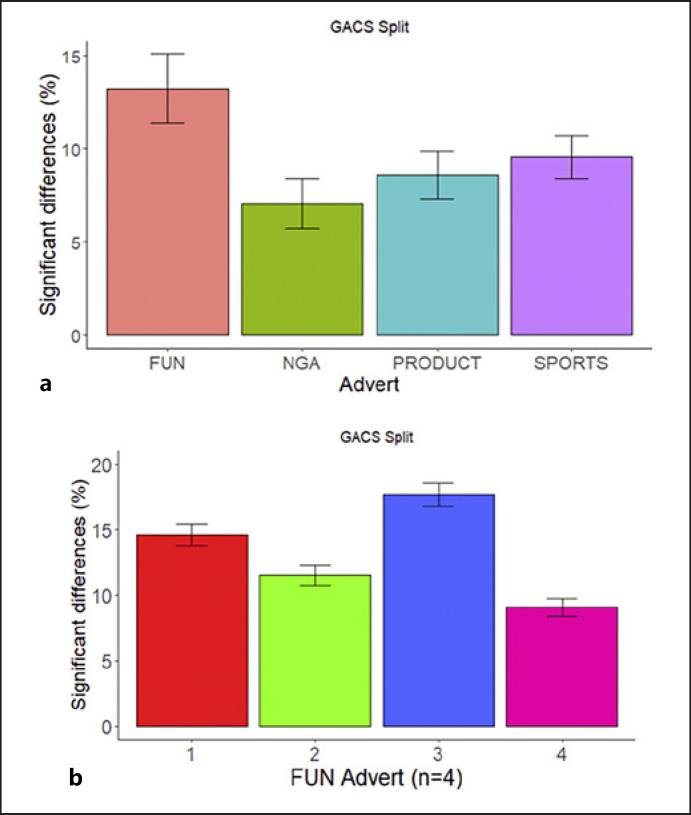
**a** Comparison of the four video categories on the basis of the percentage of frames with a significant difference (GACS split). Bars represent standard error of the mean across video clips. **b** Percentage of frames with a “significant” difference in the distances to the group centres (example based on fun adverts and a split between low and high craving participants), revealing videos with small and videos with large group differences.

The mean percentage of frames with significant differences across the four video categories is shown in Figure 4a for splits based on GACS. As shown in the figure, the videos depicting gambling as FUN (13.2 ± 1.86, mean ± SEM) show larger numbers of video frames with “significant” group differences and may therefore be best suitable to predict group membership in our sample of participants. The videos containing TV commercials (non-gambling adverts) show the smallest number of frames with “significant” group difference (7.0 ± 1.34). A comparison of the four advert clips based on GACS split revealed a non-significant effect of type of adverts on the percentage of frames with significant difference between groups, *F* (3,12) = 3.34, *p* = 0.056, η^2^ = 0.455.

### Identification of Videos with Large Difference

The error bars in Figure 4a show that there is some variability in the percentage differences per video but does not show the distribution of these differences. We therefore plot the percentage of group differences separately for each type of advert in Figure 4b, where we focus on the FUN adverts. Often the percentage of “significant” frames is around 5%, what can be expected on the basis of chance. Figure 4b shows that most videos have an average slightly above this 5%, with FUN video 1 and FUN video 3 having substantially higher percentages.

### Classifying Unseen Participants Using Machine Learning

**Fig. 5:**
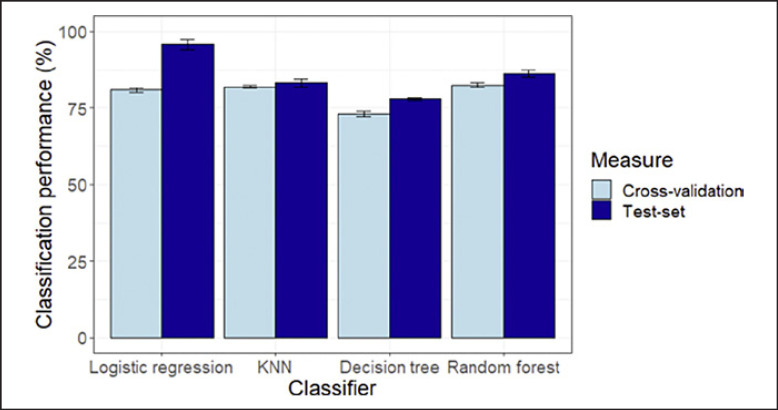
Model predictive accuracy across several classifiers for 5-fold cross-validation and test samples.

To examine whether average viewing positions for the videos with the largest group differences could predict group membership (i.e., viewers with high or low craving for gambling) on the basis of eye movements, we used different machine learning methods: a logistic regression, a KNN classifier, a decision tree, and a RF. Importantly, by splitting the data repeatedly into a training and test set examines not only how well the models can predict group membership for seen data (training set - cross validation), but also for unseen data (test set). This approach was chosen because some of these methods require selection of hyperparameters. Figure 5 shows model predictive accuracy for the different classifiers. We report here the results based on GACS split. A subscale analysis was conducted, but nothing meaningful was found (results are shown in the online suppl. material). As shown in the figure, the logistic regression yielded the best classification accuracy of 96% (close to 100%), while RFs, KNN, and decision tree achieved accuracies of 86%, 83%, and 78%, respectively, suggesting that young people's eye movement patterns across the gambling adverts could well predict their cravings for gambling.

## Discussion

We present a simple but effective data-driven method [51] to examine group differences in viewing patterns of young people who self-identify as having a low or high craving to gambling when watching gambling advertisements. Eye tracking research on gambling advertising is still relatively new, and so the methodology is not yet fully optimised. This noticeable gap in the literature is not a fault of previous studies, but rather reflects difficulties in analysing eye movement patterns for dynamic gambling ads as traditional scanpath comparison methods assume that the stimulus does not change during viewing (ScanMatch) [57]. As a result, researchers are discouraged from using such moving stimuli even though they are more ecologically valid than static images.

The method we adopted in this study was used to identify relevant videos (study aim 1) and relevant video sections (types) (study aim 2) and predict group membership on the basis of these selections of frames and videos (study aim 3). We tested how well young people's craving for gambling can be predicted from eye gaze patterns to adverts depicting gambling as fun and a sport and showing gambling products. We found that for some of the videos a substantial percentage of frames showed group differences, but that other videos have percentages near chance level and are unlikely to aid classification of participants. Generally, we found that videos depicting gambling as FUN show larger numbers of video frames with “significant” group differences and may therefore be best suitable to predict group membership in our sample of participants. The selected videos were highly effective at predicting group membership. When average fixation positions for these videos were computed for each participant and video, machine learning methods, including logistic regression and a RF, could almost perfectly predict group membership. This was not only the case for the already seen training dataset, but also for yet unseen test data.

Our findings compliment previous findings that revealed differences in gaze variability across observers [51, 58] by demonstrating differences in gaze variability between groups of participants. For instance, Dror and colleagues found that that eye movements are at least partially determined by the visual input, with gaze patterns of several observers on the same natural movie less variable than those made on different movies. Our study is similar in that differences in gaze patterns between our high and low craving groups were driven by the type of gambling adverts they watched (visual input), which in our study is those adverts which reinforced the “fun” element of participating in gambling. Our findings also add to previous research suggesting that children and young people are attracted to ads which reinforced the “fun” element of participating in gambling with low risks [10]. Perhaps those who have higher cravings might be more attracted to “fun” adverts because adverts depicting gambling as “fun” promote elements of gambling (e.g., camaraderie, winning outcomes, positive social situations, etc.) that are motivations to gamble [59].

Our study is not without limitations. First, we cannot exclude the possibility that differences in viewing patterns between low and high craving participants were exclusively due to gambling craving. Participants in the two groups may have differed in other ways, e.g., on how they would have scored on South Oaks Gambling Screen-Revised for Adolescents (SOGS-RA), a measure of problem gambling among young people [60] or in other personality constructs. Although we measured problem gambling, the uneven sample size precludes us from comparing differences in gaze patterns between those who self-identify as problem or at-risk gamblers and non-gamblers based on SOGS-RA. A follow-up study may provide more insight in whether the observed differences were solely due to gambling craving. Previous research has established that exposure to gambling advertisement increases craving [28]. Although craving was measured only once (i.e., after watching the video), it would have been pertinent to see whether craving actually increases post-exposure to gambling advertisement. Thus, future research that measures craving pre- versus post-exposure to gambling ads is needed to evaluate this possibility.

Furthermore, given that some of the videos showed a substantial percentage of frames with group differences, we cannot rule out the possibility that our findings may be limited due to low statistical power. However, given the opportunistic nature of the sample, our findings could act as a pilot finding to justify a better funded, larger-scale study to replicate our findings. As stated in the introduction, the recently developed method we adopted here is entirely data-driven and, thus, does not make inference about differences between groups, or reasons for such differences. Critically, the method differs from other data-driven methods such as saliency models [61], which test assumptions about how the brain assigns priority to different features (e.g., colour, luminance, contrast) of an image.

## Conclusion

To summarise, this is the first eye movement research in the gambling and/or addiction literature that has varied systematically different moving advertising clips in an attempt to obtain a reliable measure of the exposure to gambling advertising. With no specific experimenter provided goal, observers may cherry-pick their own idiosyncratic goals. Given that various aspects of gambling advertisements may be appealing to some viewers, it is important to begin to determine which aspects of gambling advertisements induce craving and trigger a gambling session among young people. In the present study, we present a newly developed data-driven method that helps us address some of these gaps in the literature. We demonstrated that young people's craving for gambling can be predicted based on their eye movements to video clips of gambling advertisements and that certain features of gambling advertisements may be more appealing to some group of viewers, particularly those with high craving for gambling. We encourage others to begin to apply this method to improve our understanding of the role of advertisement on gambling among teenage youths.

## Statement of Ethics

Ethics approval was gained through University of Lincoln Research Ethics Committee, approval number 2019-May-0650, and written informed consent was obtained from participants including their parents/legal guardian prior to commencement of the study. Consent was also gained from the management of the two schools where the study was conducted.

## Conflict of Interest Statement

The authors have no conflicts of interest to declare.

## Funding Sources

Funding for the study was provided by the College of Social Sciences Research, Professional Practice and Scholarly Activity Fund, University of Lincoln.

## Author Contributions

Tochukwu Onwuegbusi, Todd Hogue, and Amanda Roberts contributed to the study conception and design; Tochukwu Onwuegbusi contributed to analysis and interpretation of data; Tochukwu Onwuegbusi and Stephen Sharman drafted the manuscript; Tochukwu Onwuegbusi, Stephen Sharman, Amanda Roberts, and Todd Hogue contributed to the critical revision. All authors reviewed and approved the final version of the manuscript.

## Data Availability Statement

All data generated or analysed during this study are included in this article and its online supplementary material. Further enquiries can be directed to the corresponding author.

## Supplementary Material

Supplementary data
