# Supplementary material for: An Eye Tracking Investigation of Young People's Gaze Behaviour to Gambling and Non-Gambling Moving Adverts
Source: Eur Addict Res. 2023 Feb 7;29(2):109–18. doi: 10.1159/000529114 (PMC10273898; doi:10.1159/000529114)

**Supplementary material for: An eyetracking investigation of young people’s gaze behaviour to gambling and non-gambling moving adverts**

**Descriptive statistics**

Table 1 shows mean and standard deviation by participants gender in high and low craving group for total GCAS and each of the subscales. The result of a t-test revealed no difference between male (*M*= 17.88, *SD*=8.01) and female counterparts (*M*= 16.90, *SD*=5.80) on the total GCAS, *t* (31.33) = -0.55, *p* = .587. Also, there was no gender differences on the Anticipation, *t* (35.1) = -0.10, *p* = .919, Desire, *t* (31) = -0.49, *p*= .626 and Relief subscales, *t* (32) = -0.69, *p*=.493.

| **Table** 1. Mean and standard deviation of male and female participants in high and low craving group on the total GCAS and each of the subscales. | | | | |
| --- | --- | --- | --- | --- |
| *GCS Total* (Mean = 17.14; SD = 6.38) | | | | |
| **Gender** | **Craving split** | **N** | **Mean** | **SD** |
| Female | High | 32 | 20.38 | 7.20 |
| Female | Low | 41 | 14.20 | 1.79 |
| Male | High | 12 | 21.92 | 9.65 |
| Male | Low | 12 | 13.83 | 2.25 |
| *Anticipation subscale* (Mean = 9.66; SD =2.49) | | | | |
| Female | High | 25 | 12.12 | 1.09 |
| Female | Low | 48 | 8.35 | 1.82 |
| Male | High | 10 | 12.1 | 0.99 |
| Male | Low | 14 | 8.00 | 2.32 |
| *Desire subscale* (Mean = 3.48; SD = 2.53) | | | | |
| Female | High | 5 | 8.8 | 7.26 |
| Female | Low | 68 | 3.0 | 0.00 |
| Male | High | 3 | 9.0 | 8.66 |
| Male | Low | 21 | 3.0 | 0.00 |
| *Relief subscale* (Mean = 4.00; SD = 2.95) | | | | |
| Female | High | 13 | 7.85 | 4.88 |
| Female | Low | 60 | 3.00 | 0.00 |
| Male | High | 10 | 6.40 | 5.03 |
| Male | Low | 14 | 3.00 | 0.0 |

A description of the participants who gamble vs not in the past week, according to gender and ratings of craving is presented in Table 2.

| **Table** 2. Mean, standard deviation and number of participants who gamble in the past week by gender and ratings of craving | | | | | |
| --- | --- | --- | --- | --- | --- |
| Gender | Gambled | GCAS | N | Mean | SD |
| Female | Yes | High | 4 | 18.00 | 0.00 |
| Female | Yes | Low | 3 | 14.67 | .58 |
| Female | No | High | 28 | 20.71 | 7.65 |
| Female | No | Low | 38 | 14.16 | 1.85 |
| Male | Yes | Low | 1 | 16.00 | 0.00 |
| Male | No | High | 12 | 21.92 | 9.65 |
| Male | No | Low | 11 | 13.64 | 2.25 |

**Machine Leaning Analysis**

Figures Supp. 1 – 3 show the results of machine learning analysis for each of the three subscales. As with complete GCAS, logistic regression yielded the best classification performance compared to other classifiers for each of the three subscales (see Figures Supp 1-3).

***Figure Supp*** 1. Model predictive accuracy across several classifiers for 5-fold cross-validation and test samples (Anticipation subscale).


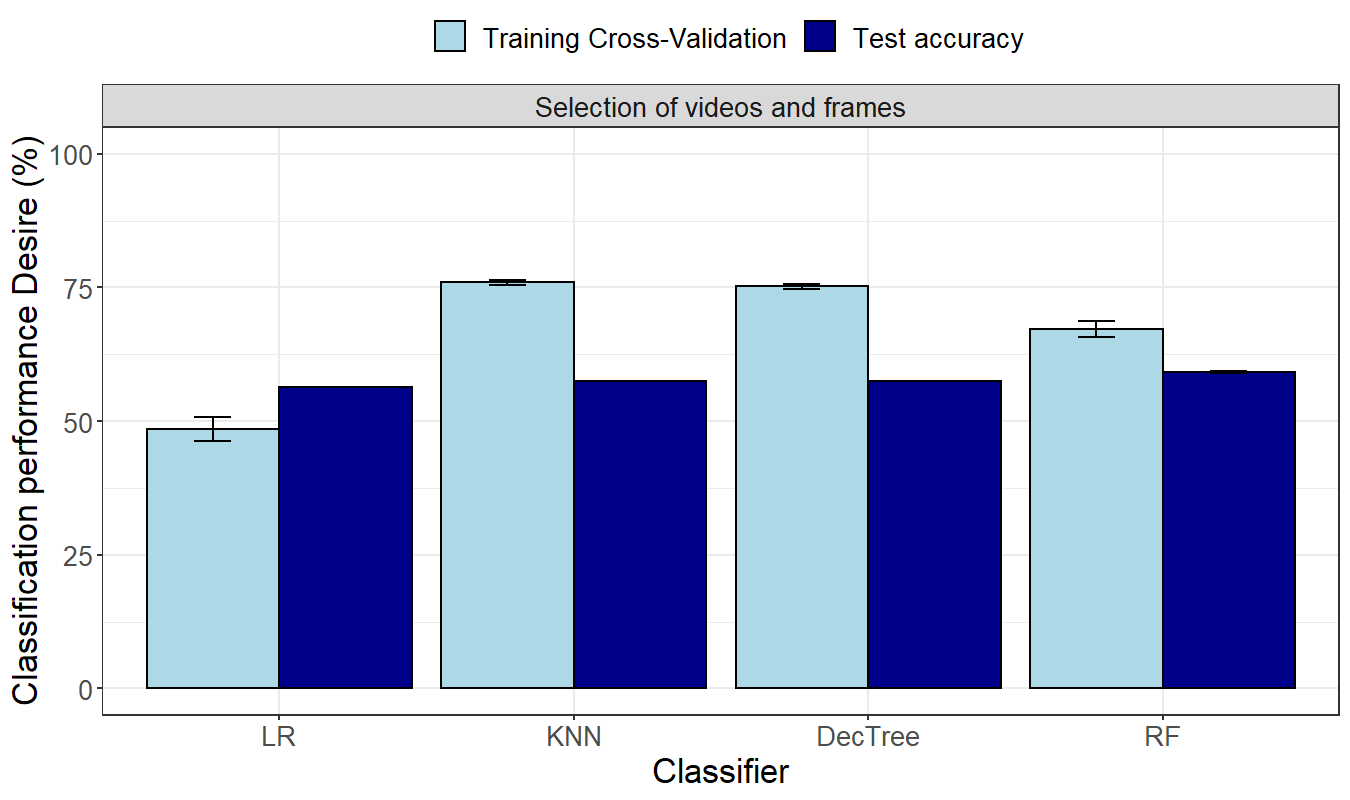


***Figure Supp*** 2. Model predictive accuracy across several classifiers for 5-fold cross-validation and test samples (Relief subscale).


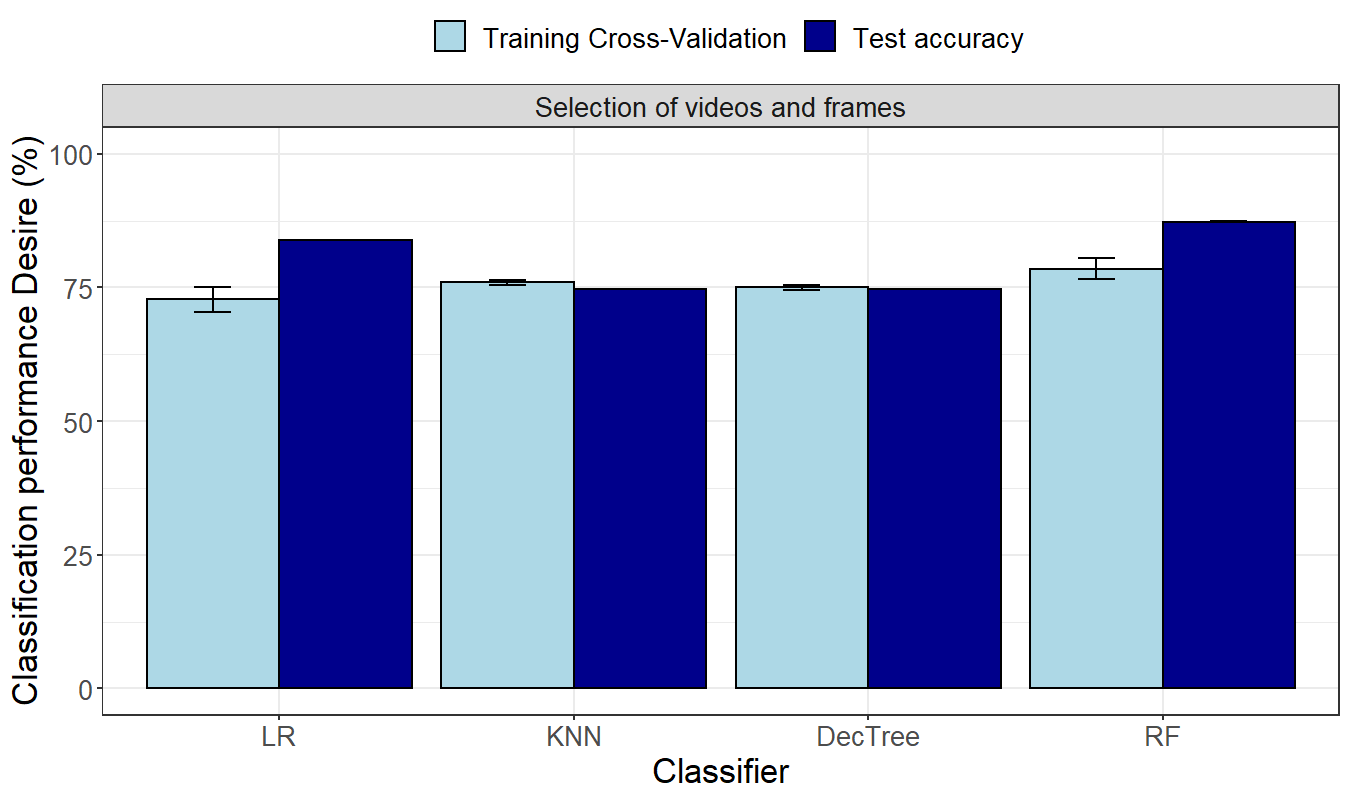


***Figure Supp*** 3. Model predictive accuracy across several classifiers for 5-fold cross-validation and test samples (Desire subscale).


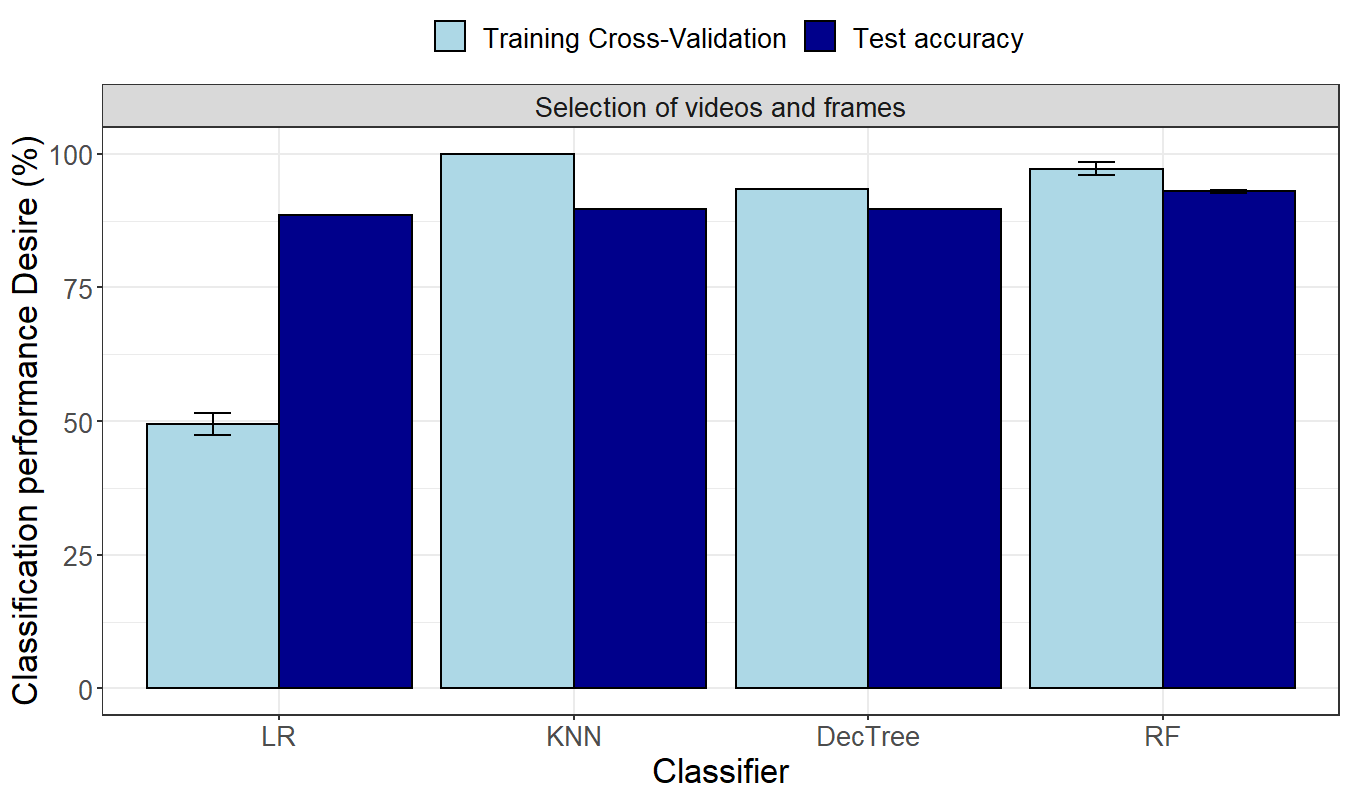

Supplement: Supplementary file 1 — Supplementary data [file ear-0029-0109-s01.docx]
